# Parasites and microorganisms associated with the snakes collected for the “*festa Dei serpari*” in Cocullo, Italy

**DOI:** 10.1371/journal.pntd.0011973

**Published:** 2024-02-21

**Authors:** Jairo Alfonso Mendoza-Roldan, Livia Perles, Ernesto Filippi, Nicole Szafranski, Gianpaolo Montinaro, Mariaelisa Carbonara, Riccardo Scalera, Pedro Paulo de Abreu Teles, Julia Walochnik, Domenico Otranto

**Affiliations:** 1 Department of Veterinary Medicine, University of Bari, Valenzano, Italy; 2 Biologist consultant for the Cocullo municipality, Rome, Italy; 3 College of Veterinary Medicine, Department of Biomedical and Diagnostic Sciences, University of Tennessee, Knoxville, United States; 4 RIFCON GmbH, Goldbeckstrasse 13, Hirschberg, Germany; 5 IUCN/SSC Invasive Species Specialist Group, Rome, Italy; 6 Center for Pathophysiology, Infectiology and Immunology, Medical University of Vienna, Vienna, Austria; 7 Department of Veterinary Clinical Sciences, City University of Hong Kong, Hong Kong, SAR China; Instituto Butantan, BRAZIL

## Abstract

While in much of the Western world snakes are feared, in the small, rural, mountainous town of Cocullo, in the middle of central Italy, snakes are annually collected and celebrated in a sacro-profane ritual. Every 1^st^ of May, *Serpari* (snake catchers) capture and showcase dozens of non-venomous snakes to celebrate the ritual of *San Domenico*. In order to detect potential zoonotic pathogens within this unique epidemiological context, parasites and microorganisms of snakes harvested for the “*festa dei serpari”* ritual were investigated. Snakes (*n =* 112) were examined and ectoparasites collected, as well as blood and feces sampled. Ectoparasites were identified morpho-molecularly, and coprological examination conducted through direct smear and flotation. Molecular screenings were performed to identify parasites and microorganisms in collected samples (i.e., Mesostigmata mites, *Anaplasma*/*Ehrlichia* spp., *Rickettsia* spp., *Borrelia burgdorferi* sensu lato, *Coxiella burnetii*, *Babesia/Theileria* spp., *Cryptosporidium* spp., *Giardia* spp., *Leishmania* spp. and helminths). Overall, 28.5% (32/112) of snakes were molecularly positive for at least one parasite and/or microorganism. Endosymbiont *Wolbachia* bacteria were identified from Macronyssidae mites and zoonotic vector-borne pathogens (e.g., *Rickettsia*, *Leishmania*), as well as orally transmitted pathogens (i.e., *Cryptosporidium*, *Giardia*, *Proteus vulgaris*, *Pseudomonas*), were detected from blood and feces. Thus, given the central role of the snakes in the tradition of Cocullo, surveys of their parasitic fauna and associated zoonotic pathogens may aid to generate conservation policies to benefit the human-snake interactions, whilst preserving the cultural patrimony of this event.

## Author summary

The “*festa dei serpari”* is a unique sacro-profane ritual held each 1^st^ of May in the small town of Cocullo, Central Italy. In this ceremony, dozens of non-venomous free-ranging snakes are captured by *serpari* (snake catchers) and showcased to thousands of pilgrims and tourists. Therefore, we aimed to assess the parasites and microorganisms of snakes within this unique epidemiological context to identify potential zoonotic pathogens. Snakes were examined and ectoparasites, blood and feces were collected, and morpho-molecular studies were performed. Overall, 28.5% (32/112) of snakes were positive for at least one parasite and/or microorganism. We identified new records of Mesostigmata mites as well as *Leishmania tarentolae* for the first time in Italian snakes. Importantly, we detected zoonotic microorganisms such as *Rickettsia* sp. in Aesculapian snake, as well as orally transmitted pathogens (i.e., *Cryptosporidium*, *Giardia*, *Pseudomonas* sp., *Proteus vulgaris*) from blood and feces of four species of snakes. Thus, snakes handled in this tradition may play a role in the zoonotic transmission of pathogens, given the contact with humans during this unique event.

## Introduction

Snakes’ (Serpentes: Squamata) perception and interaction with human societies can be contrasting, generating fear and negative feelings (e.g., disgust, repulsion; [1,2]), being merely tolerated or even used for food or economic sources (i.e., snake charming) [3], or considered as new companion animals [4,5]. One of the major threats on snake conservation is the anthropogenic pressure, directly implicated in the decline of snake populations [6,7]. Similarly, habitat and biodiversity loss and climate change represent main threats on snake populations [8–11]. Important threats are also represented by the predation by domestic carnivores (i.e., dogs and cats) and the high density of some wild ungulates, such as wild boars [12–14], as well as the emergence of pathogens (e.g., *Ophidiomyces ophidiicola*) within wild populations of snakes [15]. Both factors above are connected, as many pathogens are transmitted by predation, with snakes being intermediate or definitive hosts of parasites, some of which are zoonotic [16].

The relationships and uses by human communities of reptiles is also known as ethnoherpetology [17], which studies the importance of reptiles in different ecological, economic, and cultural contexts [2]. Further investigations were conducted toward integrating One-Health parasitological approaches with ethnoherpetology (i.e., ethnoherpetoparasitology), which allowed to identify the microorganisms and parasites that these animals harbor, as well as the potential risk of zoonotic transmission to snake charmers and vendors in the souks of Marrakech, Morocco [3]. All of the above was assessed in a place where snakes are feared but highly tolerated, given their cultural and economic importance.

In Italy, these slithery animals are part of socio-cultural and religious aspects of the country’s history. One of the most ancient and iconic ethnoherpetological rituals across Europe, known as the *“festa dei serpari”* (also called the ritual of *San Domenico*), is performed in the small mountainous town of Cocullo, central Apennine (Abruzzo, central Italy) [6,18,19]. This mixed Catholic and pagan ceremony has been performed for centuries during the first days of May, with little to no alterations, consisting on placing four-lined snakes (i.e., *E*. *quatuorlineata*) on top of the statue of *San Domenico* [20]. Soon after, the snake-adorned statue is taken through the small town, in a religious procession with thousands of onlookers in attendance. During the main event, other species of snakes (e.g., western whip snake—*Hierophis viridiflavus*, Aesculapian snake—*Zamenis longissimus*, juvenile specimens of *E*. *quatuorlineata*) are handled by “*serpari”* (i.e., people that capture and handle the snakes) for thousands of pilgrims and tourists to photograph or interact with them [18,21]. In order to have a good number of snakes for the festival, the “*serpari”* are formally authorized by relevant authorities to capture snakes alive in the surroundings of the Cocullo municipality from the 19^th^ of March till the 30^th^ of April, after which they are obliged to release them in the same capture sites, within three days following the main event.

In Italy, most parasitological studies on snakes have been focused on *Cryptosporidium*, helminths and ectoparasites in exotic/pet snakes [22–24], with few investigations focused on wild species [25]. In the same context, the introduction of exotic parasites (e.g., *Renifer aniarum*) in grass snakes (*Natrix natrix*) and of snake fungal disease (SFD) caused by *O*. *ophidiicola* in dice snakes (*Natrix tessellata*) stresses the importance of monitoring the health status of wild populations of animals by accurate risk assessment [26–28]. In addition, aside from salmonellosis [29,30], zoonotic parasites of reptiles [16,31], including Reptile Vector-Borne diseases (RVBDs; [32]) have gained interest of the scientific community. Indeed, wild snakes are sentinels for zoonotic agents as they are reservoirs of a plethora of pathogens, playing a role in the life cycle of helminths (i.e., cestodes, nematodes and trematodes), pentastomids and vector-borne pathogens [16]. Ticks such as *Ixodes ricinus* have been collected from wild four-lined snakes (*Elaphe quatuorlineata*) from southern Italy, that tested positive for Mediterranean spotted-fever (*Rickettsia helvetica*), but not for Lyme disease (*Borrelia burgdorferi* sensu lato [33–35]). Other zoonotic parasites have been identified in free-ranging snakes, such as *Spirometra erinaceieuropaei*, *Mesocestoides* and *Raillietiella* [25,36]. Moreover, efforts have been carried out to assess possible emerging pathogens that could be a threat to the snake populations, such as bacteria [37], and the devastating keratinophilic fungus *O*. *ophidiicola* [27]. Considering all the above, the present study aimed to investigate parasites and microorganisms associated to snakes collected for the “*festa dei serpari”* ritual, as well as to identify potential zoonotic pathogens that these animals may harbor.

## Methods

### Ethics statement

The study was conducted in accordance with all applicable international, national, and/or institutional guidelines for the care and use of animals. One of the major goals was to monitor for threats that could negatively impact the health of snakes due to their handling and exposure to humans within the ritual, whilst continuing to preserve the cultural patrimony of this event. Protocols of snake sampling, handling, and capture by *Serpari*, as well as by scientific committee, is allowed under the National authorizations (National law DPR 357/97). The permit was granted by the Italian Ministry of Environment (n. 16271/2023 PNM and 79052/2023 PNM).

### Animal examination and sampling

Given its cultural, religious, and historical importance, the ritual is accredited by local authorities, which in recent years have worked on increasing the awareness of the importance of conservation of the snake populations to perpetuate the traditional ritual. Since 2010, all “*serpari”* must declare captured snakes to a scientific committee established specifically for the purpose of the event by the major of the town (EF; GM). The scientific committee is in charge of the operations to record all captured animals along with their biometric data (i.e., weight, snout-vent length measures), and other details, such as sex, age class (juvenile, subadult, adult), and site of capture. The sex of snakes was determined by probing, using round-ended metallic probes, or palpating the hemipenes. Additionally, animals are marked with a subcutaneous Passive Integrated Transponder (PIT) tag, which allows to recognize animals in case of recaptures. Moreover, the scientific committee includes a veterinarian that performs clinical exams on all animals. This initiative has improved the welfare conditions of the collected snakes, as well as educated the *“serpari”* on animal husbandry. The added value of the presence of the scientific committee has permitted scientists to improve the knowledge of the distribution of different snake species at local and regional scale and to assess the snake population dynamics, accounting for more than 1300 animals examined to date [27].

**Fig 1:**
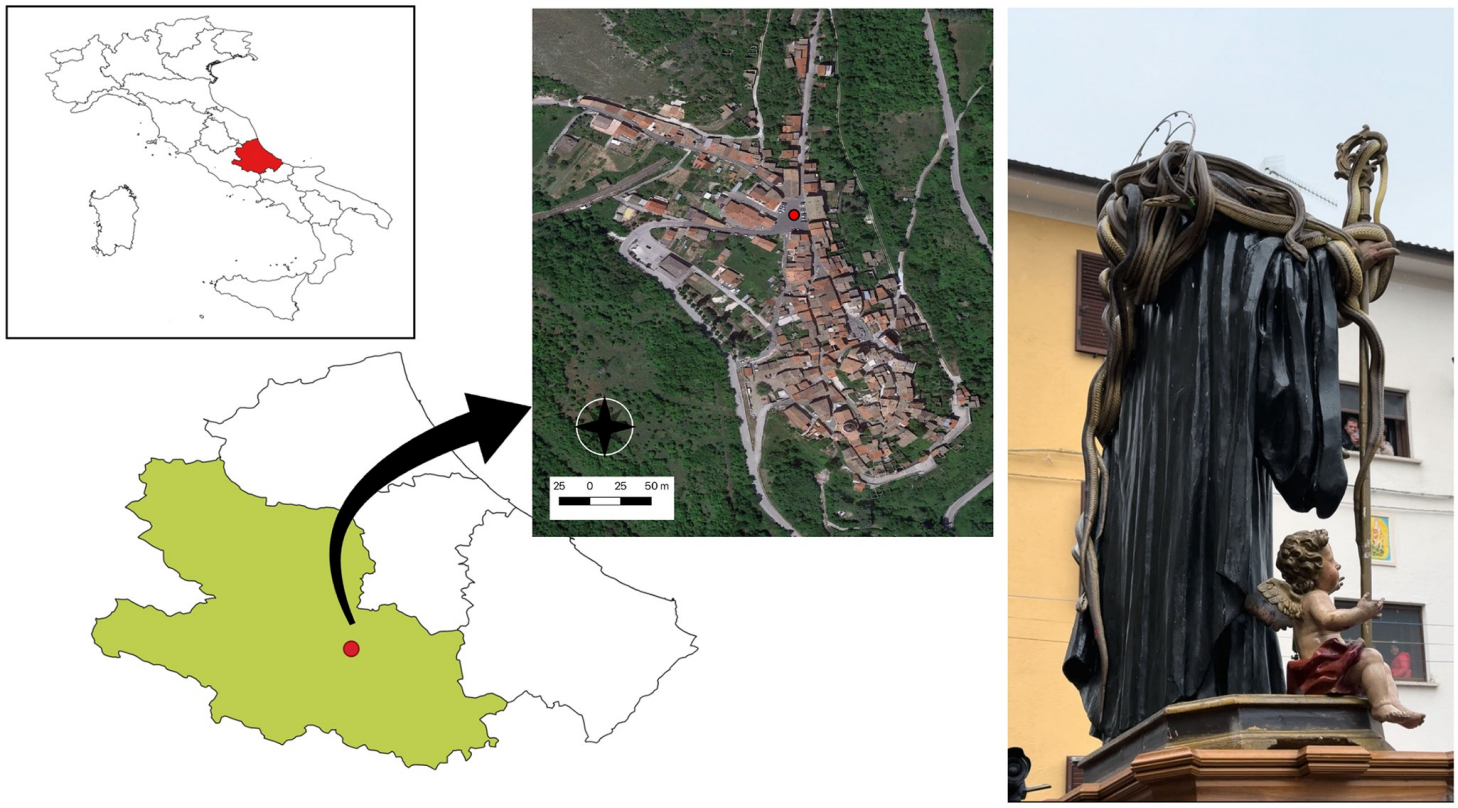
Map of the town of Cocullo, Abruzzo, Central Italy, where the ritual of *“festa dei serpari”* is celebrated. L’Aquila province is evidenced in light green. Red circles indicate the municipality of Cocullo, as well as the town square where the statue of San Domenico is covered in snakes. Map prepared using QGIS software—Buenos Aires version (link of the XYZ tile: https://gdg.sc.egov.usda.gov/GDGOrder.aspx).

**Fig 2:**
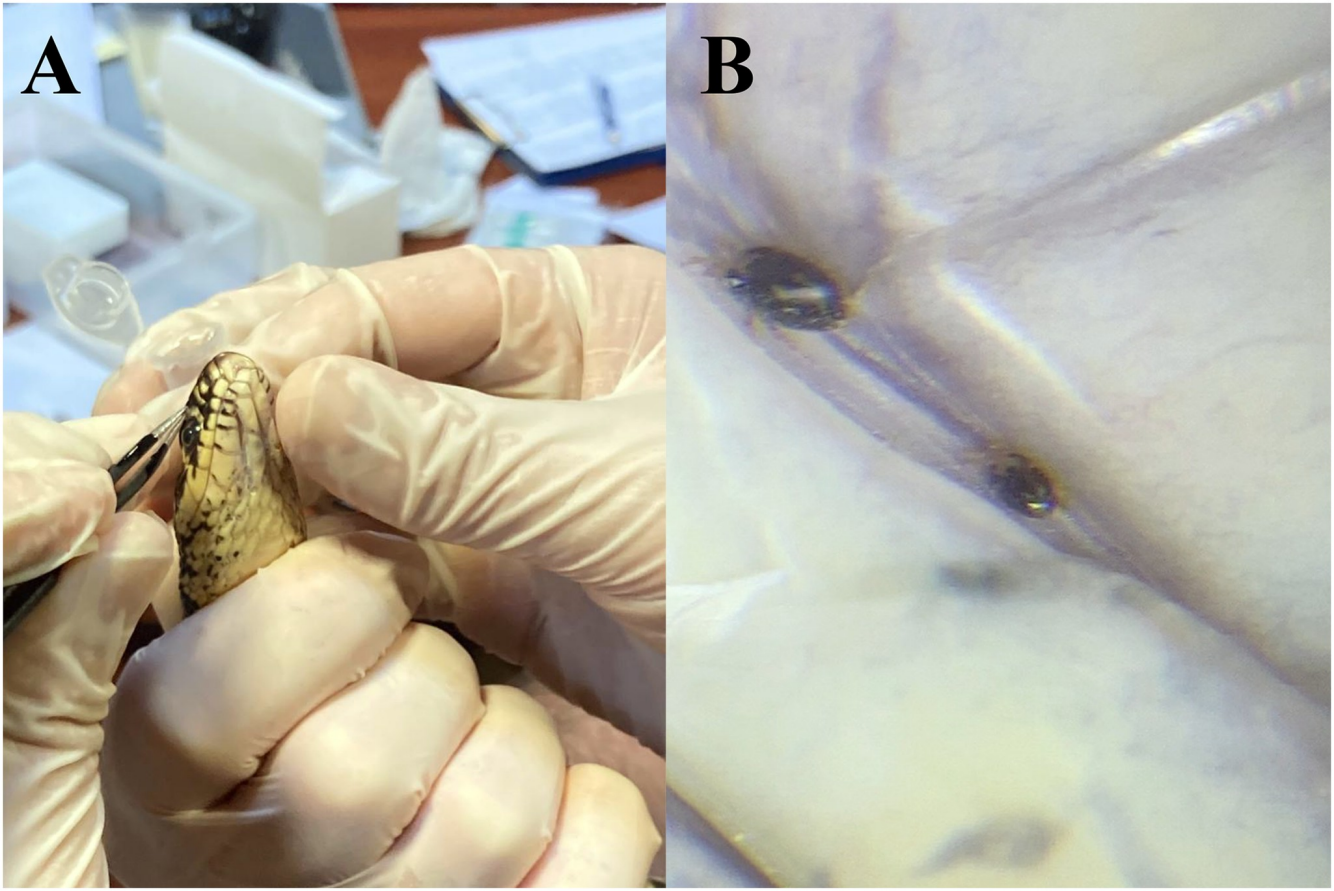
Ectoparasite collection from snakes. a) mite collection from the gular region from a western whip-tail snake (*H*. *viridiflavus*); b) Macronyssidae mites in the gular area of a whip-tail snake (*H*. *viridiflavus*).

**Fig 3:**
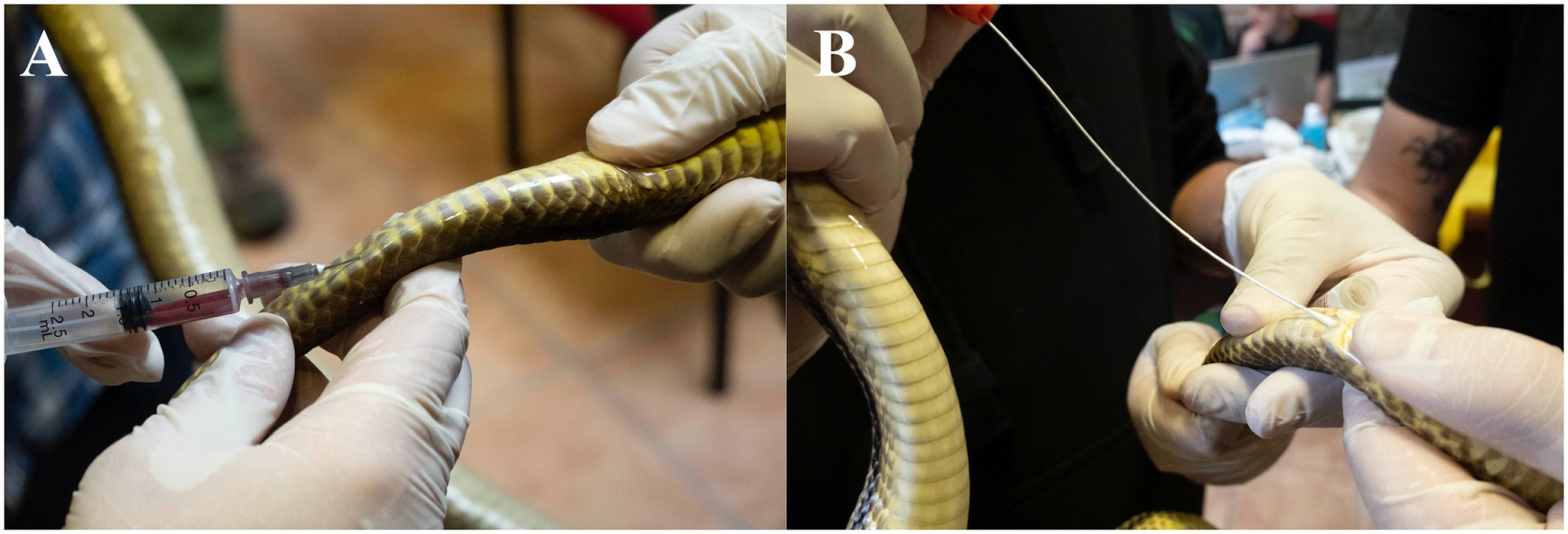
Blood and fecal sampling of snakes. a) blood draw from the ventral coccygeal vein from a four-lined snake *(E. quatuorlineata);* b) cloacal swab from a four-lined snake (*E. quatuorlineata*).

Snakes captured for the “*Festa dei serpari”* event in Cocullo, Abruzzo (Fig 1) were examined the 29^th^ and 30^th^ of April 2023, as part of the annual monitoring program performed by the local authorities and a scientific committee (EF, GM). Animals were examined according to the protocols of the monitoring program described elsewhere [27]. After these procedures, animals were clinically assessed and examined for ectoparasites and when found they were removed by scarification methods and stored in 70% ethanol (Fig 2). Blood samples (~100 μl to 1 ml; Fig 3A) were drawn from all animals using the ventral coccygeal vein. Blood was divided between Whatman FTA Cards and 1.5 ml *Eppendorf* tubes which were later stored at -20°C. Blood smears were performed from all animals and then assessed for the presence of hemoparasites [38] using Diff-Quik stain [39], and later evaluated using an optical microscope (LEICA DM LB2, Germany). Cloacal swabs (Fig 3B) were performed from all animals and stored at -20°C. Whenever possible, fecal samples were collected and stored in 1.5 ml *Eppendorf* tubes at 4°C.

### Ectoparasite processing and identification

Ectoparasites were slide-mounted in Hoyer’s medium [40] and identified using dichotomous keys [41–43], as well as original species descriptions [42,44] were used for morphological identification of Mesostigmata mites. To assess the parasitic load of mites, descriptive statistics was calculated using Quantitative Parasitology software, version 3.0 [45]. Prevalence, mean abundance (i.e., number of mites per total number of hosts) and mean intensity (i.e., number of mites per number of infested hosts) were calculated.

### Coprological studies

Fecal samples were stored at 4°C and analyzed within 48 hours. Due to the low volume obtained per individual snake (~50 μl), all samples were only analyzed microscopically through direct smear (using saline solution) to observe motile protozoa, helminths, acanthocephalans, and pentastomids, as well as a flotation test with a low-density solution was performed (saturated ZnCl solution, specific gravity 1350) [23].

### Molecular screening

DNA of mites was extracted via lysis using the guanidine isothiocyanate protocol (GT) [46]. This protocol was adapted to avoid mite destruction, which allowed the preservation of a voucher for morphological evaluation [33]. Extractions were performed from individual mites. DNA was extracted from individual blood samples (*n* = 112), feces (*n* = 38), and cloacal swabs (*n* = 101) using commercial kits (QIAamp DNA Mini Kit and DNeasy PowerSoil kit, Qiagen, Hilden, Germany), according to the manufacturer’s instructions.

PCRs of the mites were performed to confirm species identity using two molecular markers: Cytochrome Oxidase subunit 1 (*cox*1; primers Cox1 LCO1490 and HCO2198), that amplifies 680 bp fragment [47], and primers for the 18S rRNA gene (18S+ and 18S−, respectively), which amplify a fragment of 480 bp of the V4 region [48]. Cycling conditions for both PCRs were initial denaturation at 94°C for 1 min, then 30 cycles of 20 s at 94°C, 50°C for 30 s and 72°C for 1 min and 30 s, with a final extension of 72°C for 7 min.

DNA extracted from mites, blood, feces, and cloacal swabs was analyzed for the detection of different microorganisms and parasites through cPCR and qPCR protocols (Table 1). All cPCR products were examined on 2% agarose gel stained with GelRed (VWR International PBI, Milan, Italy) and visualized on a GelLogic 100 gel documentation system (Kodak, New York, USA). Amplicons were then purified and sequenced in both directions using the same PCR primers, by the Big Dye Terminator version 3.1 chemistry in a 3130 Genetic Analyzer (Applied Bio-systems, Foster City, CA, USA). Sequences were edited and analyzed using Geneious Prime software version 9.0 (Biomatters Ltd., Auckland, New Zealand) [49] and compared with those available in the GenBank database by the Basic Local Alignment Search Tool (BLAST; http://blast.ncbi.nlm.nih.gov/Blast.cgi) for species identification.

**Table 1:** Pathogens screened in this study by conventional (c) and quantitative (q) PCR, with target genes, primers, probes nucleotide sequences and fragment length.

	Pathogens	Target gene	Primers	Sequence (5′−3′)	Fragment length (bp)	References
**cPCR**	*Anaplasma*/*Ehrlichia* spp.	16S rRNA	*EHR-16SD*	GGTACCYACAGAAGAAGTCC	345	[50]
			*EHR-16SR*	TAGCACTCATCGTTTACAGC		
	*Borrelia burgdorferi* sensu lato	Flagellin	*FLA1*	AGAGCAACTTACAGACGAAATTAAT	482	[51]
			*FLA2*	CAAGTCTATTTTGGAAAGCACCTAA		
	*Rickettsia* spp.	*glt*A	*CS-78F*	GCAAGTATCGGTGAGGATGTAAT	401	[52]
			*CS-323R*	GCTTCCTTAAAATTCAATAAATCAGGAT		
	Spotted Fever Group Rickettsiae	*omp*A	*Rr190*.*70F*	ATGGCGAATATTTCTCCAAAA	632	[53]
			*Rr190*.*701R*	GTTCCGTTAATGGCAGCATCT		
	*Coxiella burnetii*	IS1111a	Trans-1	TATGTATCCACCGTAGCCAGT	687	[54]
			Trans-2	CCCAACAACACCTCCTTATTC		
	*Babesia/Theileria* spp.	18S rRNA	RLB-F	GAGGTAGTGACAAGAAATAACAATA	460–520	[55]
			RLB-R	TCTTCGATCCCCTAACTTTC		
	Cestodes/Nematodes	*cox*1	JB3	TTTTTTGGGCATCCTGAGGTTTAT	400	[56]
			JB4.5	TAAAGAAAGAACATAATGAAAATG		
	*Leishmania* spp.	ITS1	L5.8S	TGATACCACTTATCGCACTT	320	[57]
			LITSR	CTGGATCATTT-TCCGATG		
	Trypanosomatidae	18S rRNA	18SN1F	GGATAACAAAGG AGCAGCCTCTA	332	[58]
			18SN1R	CTCCACACT TTG GTTCTTGATTGA		
**qPCR**	*Leishmania* spp.	kinetoplast	LEISH-1	AACTTTTCTGGTCCTCCGGGTAG	120	[59]
			LEISH-2	ACCCCCAGTTTCCCGCC		
			Probe	6-FAM-AAAAATGGGTGCAGAAAT-MGB		
	Duplex *Leishmania*	ITS1	L.i.t. -ITS1-F	GCAGTAAAAAAAAGGCCG	150	[60]
			L.i.t. -ITS1-R	CGGCTCACATAACGTGTCGCG		
			Probe L.t.	6-FAM-CACGCCGCGTATACAAAAACAC-MGB		
			Probe L.i.	VIC-TAACGCACCGCCTATACAAAAGCA-MGB		
	*Giardia duodenalis*	SSU	Giardia-80F	GACGGCTCAGGACAACGGTT	62	[61]
			Giardia-127R	TTGCCAGCGGTGTCCG		
			Giardi-105	Fam-5′- CCCGCGGCGGTCCCTGCTAG-3′-Tamra		

Additionally, fecal samples and cloacal swabs were tested using a multiplex (5plex) qPCR for *Blastocystis hominis*, *Cryptosporidium* spp., *Dientamoeba fragilis*, *Entamoeba histolytica*, and *Giardia duodenalis* assemblages A and B [62]. In order to have sequences of the positive samples, nested PCRs were performed for *Giardia* spp. and *Cryptosporidium* spp. as follows. For *Giardia*, a nested PCR amplifying a partial sequence of the triosephosphate isomerase (*tpi*) gene (532 bp) was used that detects all known assemblages [63, 64]. For *Cryptosporidium* spp., a nPCR targeting a fragment of the 18S rRNA gene was run [65].

### Phylogenetic analyses

Mite 18S rRNA and Rickettsial *gltA*, were separately aligned against those closely related species available from GenBank database using the ClustalW application within MEGA7 software [66]. The Akaike Information Criterion (AIC) option in MEGA7 was used to establish the best nucleotide substitution model adapted to each sequence alignment. Tamura 3-parameter model with invariant sites (I) [66] was used to generate the *gltA* trees and Tamura 3-parameter model with invariant sites (I) and discrete Gamma distribution (G) for 18S rRNA of mites, and Kimura 2-parameter model with invariant sites (I) and discrete Gamma distribution (G) for 18S rRNA of *Leishmania*. Maximum likelihood (ML) phylogenetic inference was used with 2000 bootstrap replicates to generate the phylogenetic tree in MEGA7. Homologous sequences of 18S rRNA for *Ixodes ricinus* tick (Z74479) were used as outgroup to root the trees, as well as for *Rickettsia* including the *glt*A sequences from *Rickettsia belli* and *Rickettsia canadensis* (AB297809), and the 18S rRNA sequence of sequence of *Trypanosoma brucei* (XR_002989995).

## Results

Overall, 112 snakes were examined and screened representing two families and five species (Table 2). Only two animals were not marked with PIT tags because of their small body size (one *Coronella girondica*, one *Zamenis longissimus*). Most individuals were apparently healthy, with three animals having some type of external lesion (Table 2).

**Table 2:** Species of snakes (scientific and common names) sampled, sex and clinical observations.

Family	Species	Common name	*n*	Sex	Observations
Colubridae	*Coronella girondica*	Southern smooth snake	1	Female (1)	
	*Elaphe quatuorlineata*	Four-lined snake	66	Male (47) Female (19)	Abscesses of masses in their dorsal subcutaneous area (2)
	*Hierophis viridiflavus*	Western whip snake	28	Male (20) Female (8)	Skin lesions compatible scale loses due to trauma (1)
	*Zamenis longissimus*	Aesculapian snake	15	Male (20) Female (8)	
Natricidae	*Natrix helvetica*	Grass snake	2	Female (2)	
Total			112	Male (87) Female (38)	

Of the 101 blood smears performed and examined, none of them had visible hemoparasites. Additionally, ectoparasites were collected from 10.7% of snakes (12/112) being all of them mites (*n* = 46) of the Mesostigmata order. Species of mites, their sex and snake hosts, as well as infestation rates are summarized in Table 3. Namely, mites were identified as *Hemilaelaps piger* (Berlese, 1918) (Ixodorhynchidae: Mesostigmata) in two *H*. *viridiflavus*, one of them also co-infested with the second species identified, *Ophionyssus* sp. (Macronissydae: Mesostigmata). The latter mite species was identified in the remaining 11 infested snakes (Table 3). Mites were found mainly in the gular area (Fig 2B).

**Table 3:** Mite species, snake host, biological stage (nymph = N; male = M; female = F), infestation rates and sequence accession numbers (AN).

Mites Species	Snake species	*N* snakes	*n* and sex of mites	Prevalence	Mean Intensity	Mean Abundance	Sequence AN
*Hemilaelaps piger*	*Hierophis viridiflavus*	2*	5F	1.8% (2/112)	2.5 (95% CI: 1–2.5)	0.37 (95% CI: 0.16–0.79),	18S rRNA: OR771478, OR771479
*Ophionyssus* sp.	*Elaphe quatuorlineata*	3	2M; 1F	9.8% (11/112)	3.73 (95% CI: 2–6.55)	0.04 (95% CI: 0.0–0.16)	18S rRNA: OR771465
	*Hierophis viridiflavus*	8*	1N; 3M; 32F				18S rRNA: OR771463 *cox*1: OR763078
	*Zamenis longissimus*	1	2F				18S rRNA OR771469
**Total**		12	1N; 5M;35F (46)				

*one snake co-infested

**Fig 4:**
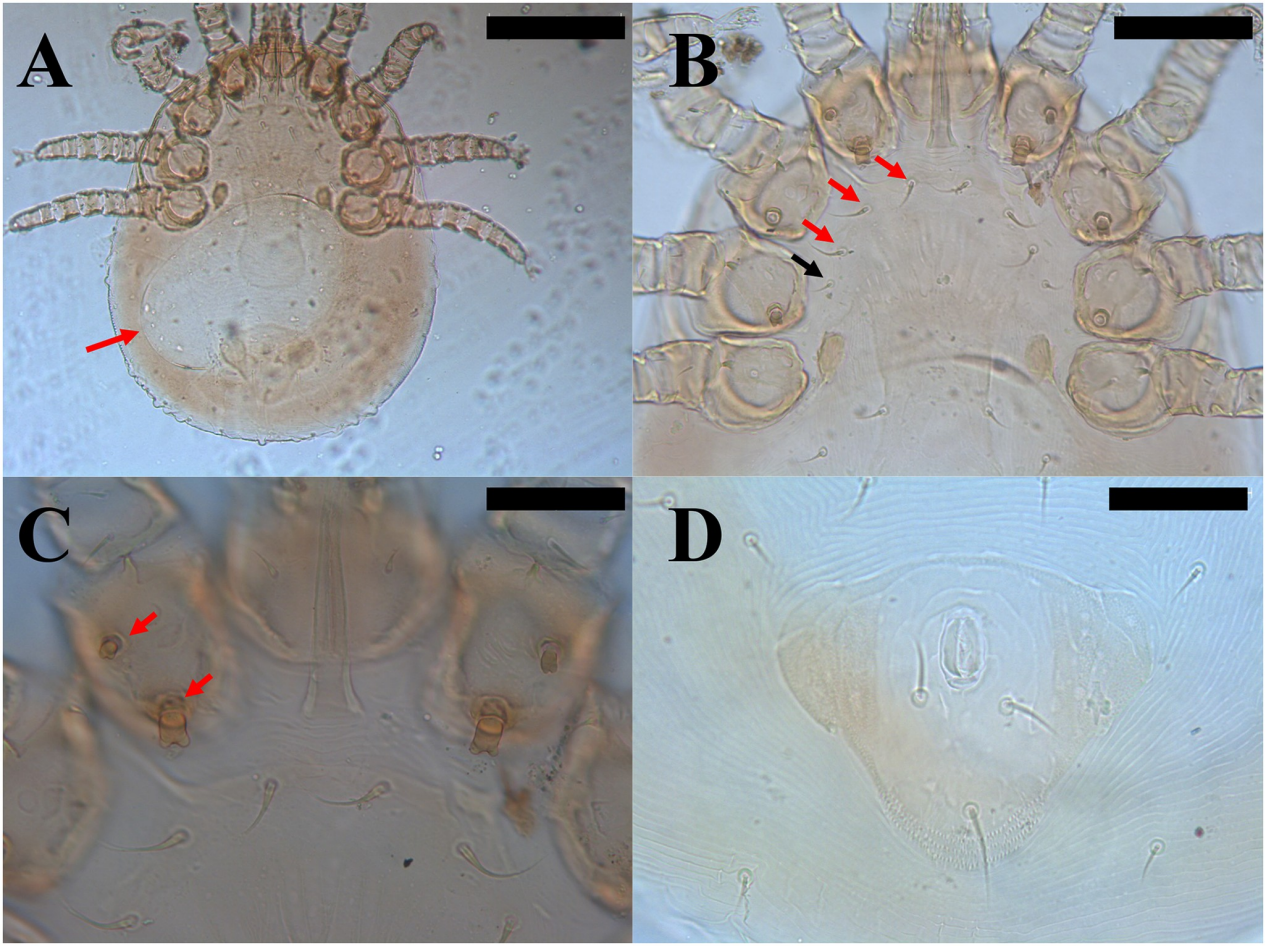
Morphological features of *Hemilaelaps piger* females. a) egg in the idiosoma of a female (red arrow); b) Three pairs of sternal setae (red arrows) and one pair of metasternal setae (black arrow); c) coxae I bear two strong bifid spurs (red arrows) typical of the *piger* group; d) the anal shield within a *cribrum*. Scale bars: 200μm (a); 100μm (b); 50μm (c,d).

*Hemilaelaps piger* were all females (*n* = 5) and some of them bearing eggs (Fig 4A). Morphologically, mites displayed features such as three pairs of sternal setae (Fig 4B) and one pair of metasternal setae. The species identified in this study belongs to the *piger* group, given that all coxae I bear two strong bifid spurs (Fig 4C), and coxae II and III have a single bifid spur and a simple seta. Observed females had dorsal shield bearing 34–36 setae. The sclerotized part of the sternal shield was short, arched and very broad, the anal shield is within a *cribrum* (Fig 4D), and the anal opening is in the anterior part of the anal shield. Both 18S gene sequences obtained herein had nucleotide identity of 99.2% with sequences of *Hemilaelaps triangulus* from captive snakes of Mexico (i.e., MT163322, MT163324, MT163324).

**Fig 5:**
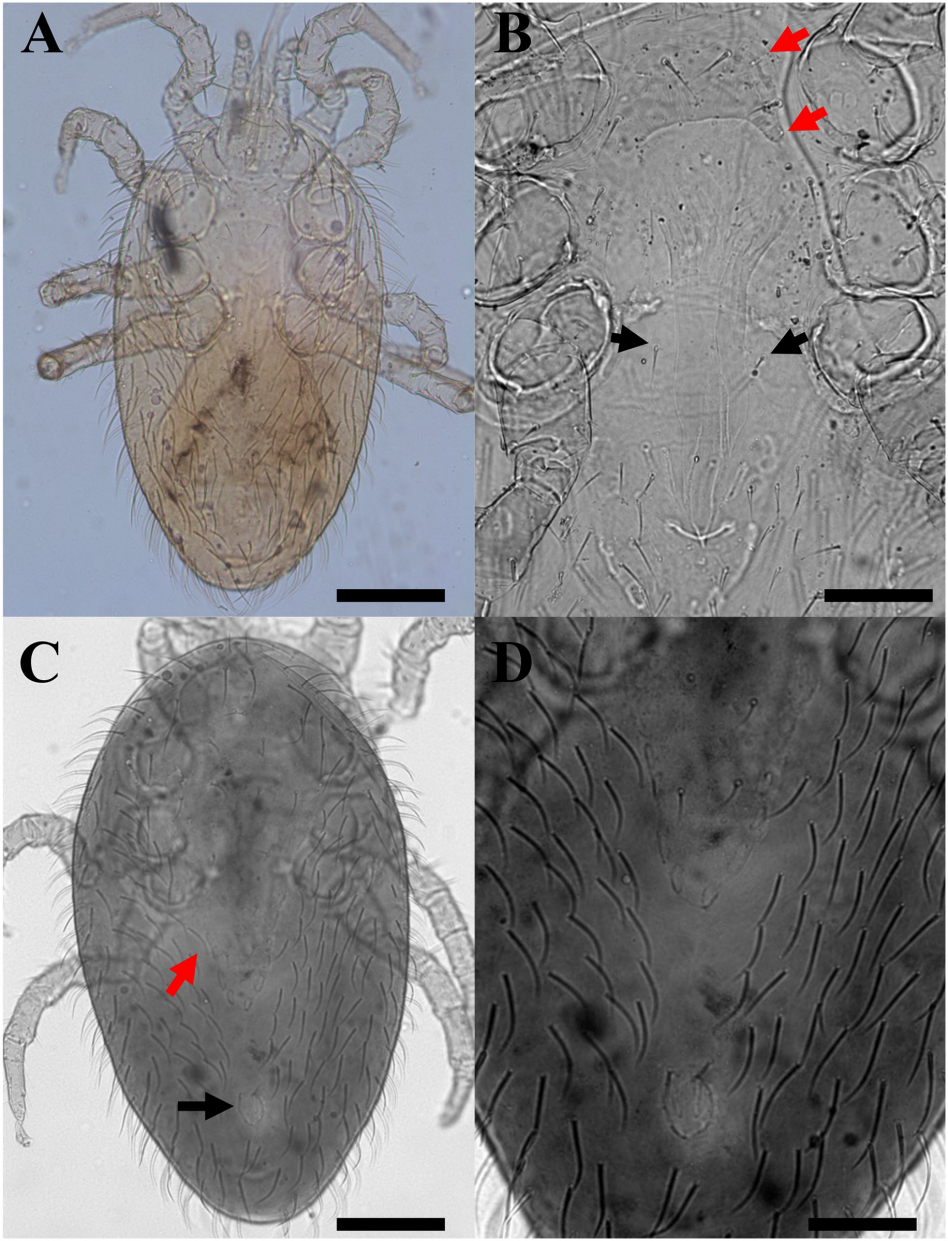
Morphological features of *Ophionyssus* sp. a) dorsal view of female *Ophionyssus* sp; b) pore pairs on sternal shield (black arrows); c) dorsal shield divided in a large anterior podonotal shield (red arrow) and a small pygidial shield (black arrow); d) absence of mesonotal scutae in between the podonotal and pygidial shield. Scale bars: 200μm (a,c); 50μm (b, d).

**Fig 6:**
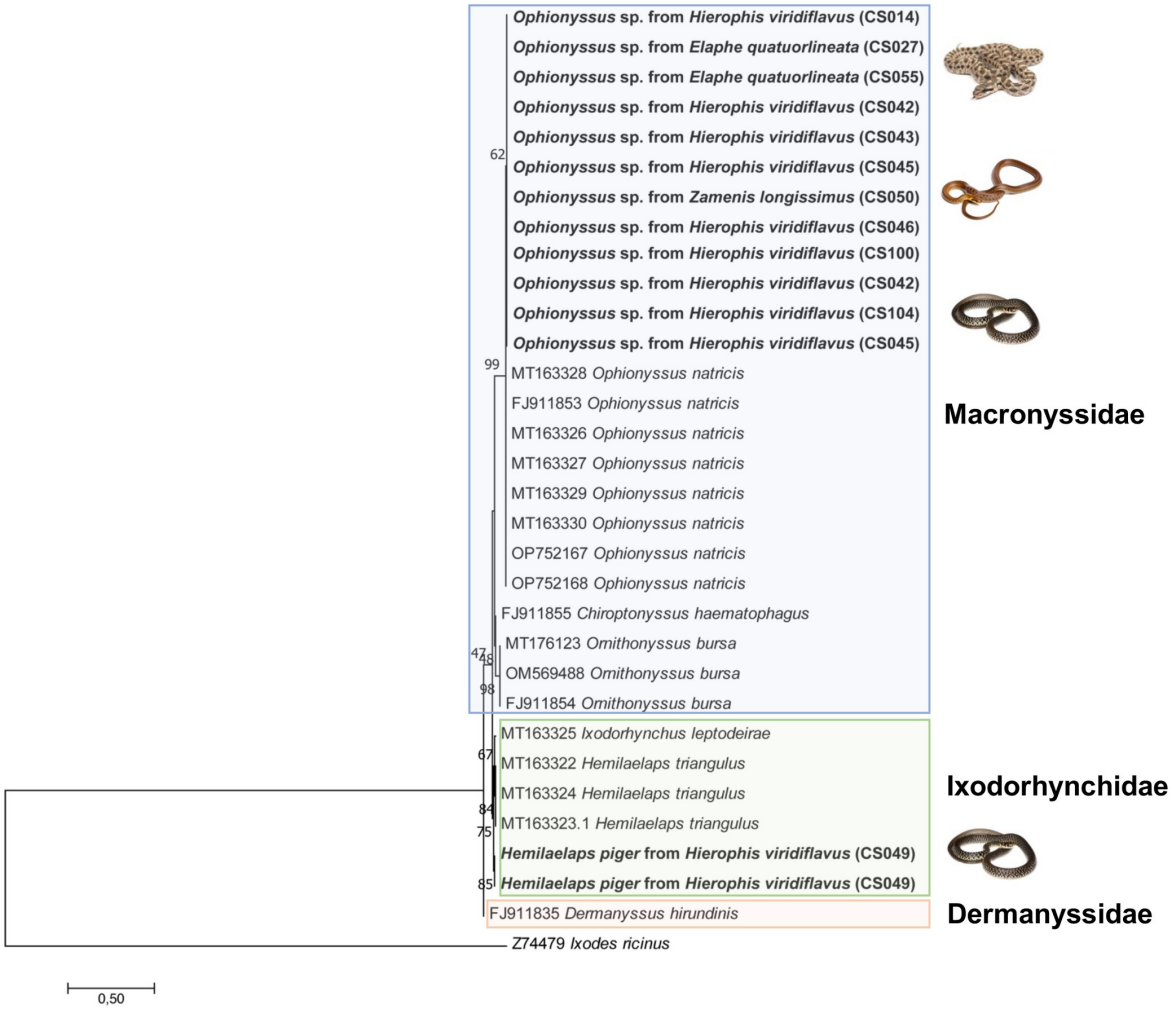
Maximum-likelihood phylogenetic trees of 18S rRNA genes of Mesostigmata mites. Bootstrap values (>40%) are shown near the nodes. *Ixodes ricinus* was used as outgroup. Scale bar indicates nucleotide substitution per site. Sequences of this study are in bold.

The remaining mites (*n =* 41) were all identified as *Ophionyssus* sp. (Fig 5A), most of them females (*n =* 32), characterized by having less than three pore pairs on sternal shield (Fig 5B). Also, the Genu III have 10 setae, and the epigynal shield is surrounded by the genital setae inserted on the integument (Fig 5B). Moreover, females had a dorsal shield divided in a large anterior and a small pygidial shield (Fig 5C) but differed in not having mesonotal scutae (Fig 5D) and having 9 pair of setae in the podonotal shield. Additionally, sequences obtained from 18S (*n =* 14) had high nucleotide similarities (99.7%) with *Ophionyssus natricis* from captive snakes of Italy (OP752167). On the other hand, *cox*1 sequences (*n =* 15) had low homology (85.8%) with those of *O*. *natricis* from captive snakes in Mexico (i.e., MT154424, MT154425). The 18S rRNA ML tree clustered the 13 generated sequences of *Ophionyssus* sp. with the available sequences of *Ophionyssus natricis*, with high bootstrap values (99%). On the other hand, the two sequences of *H*. *piger* clustered together with sequences of *H*. *triangulus* and *Ixodorhynchus leptodeirae* (Fig 6). Representative sequences herein generated were deposited in GenBank (Table 3).

**Fig 7:**
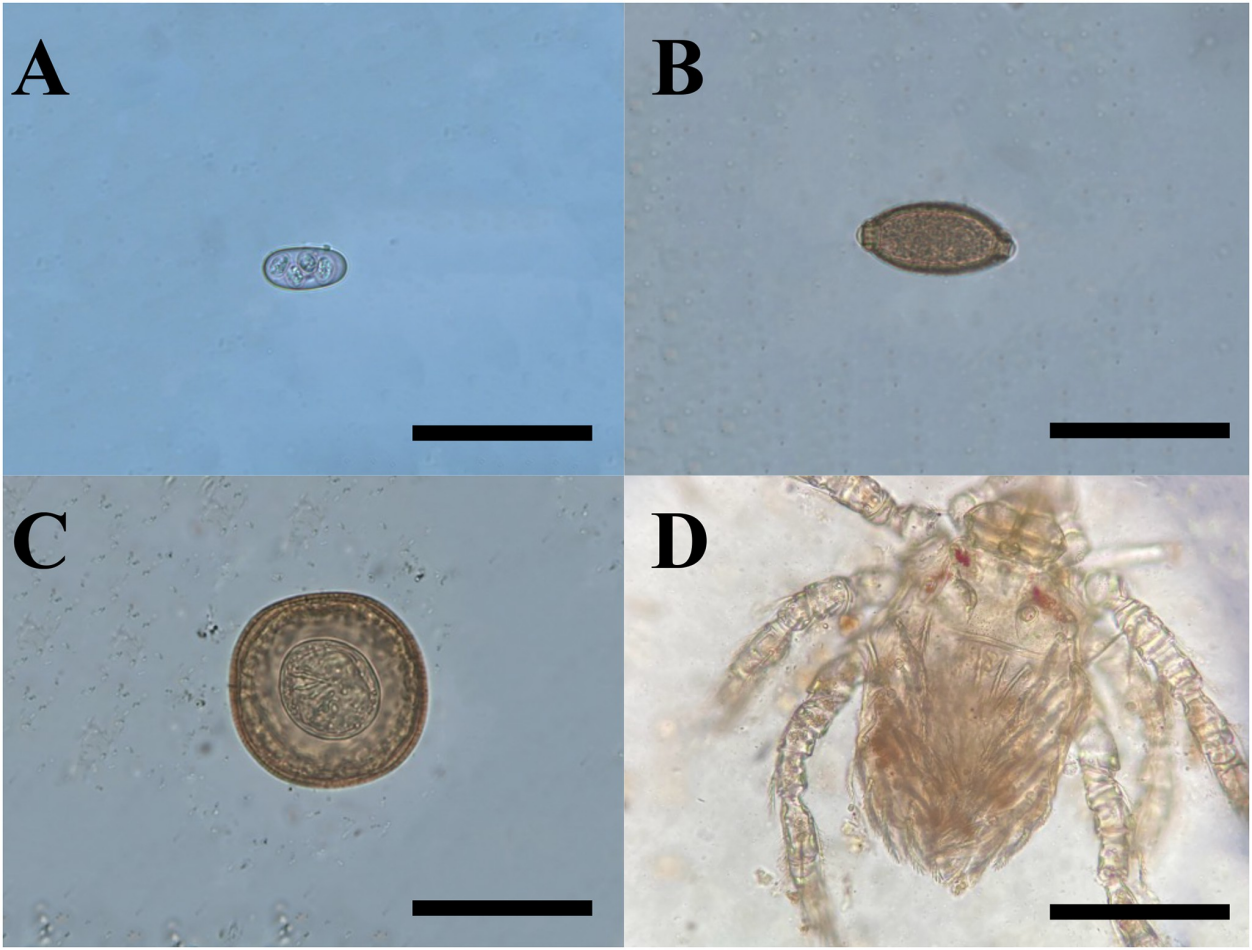
Parasitic forms found in coprological exams *H*. *viridiflavus*. a) sporulated *Eimeria* sp.; b) Capillarid egg; c) Taeniid egg; d) pseudoparasite Trombiculidae mite larva from. Scale bars: 100μm (a); 200μm (b,d); 20μm (c).

Furthermore, of the 38 fecal samples collected from different snake species (i.e., 16 *H*. *viridiflavus*, 10 *E*. *quatuorlineata*, 2 *Natrix helvetica*, and 10 *Z*. *longissimus*), 39.4% (15/38) scored positive for parasites, with 26.3% (10/38) positive at direct fecal examination, and 13.1% (5/38) through flotation test, being only two positive for both tests (i.e., two *H*. *viridiflavus*; Table 4). Briefly, ciliates (i.e., *Nyctotherus* sp.) were observed in two *H*. *viridiflavus* and Coccidia (Fig 7A) were detected in two *H*. *viridiflavus*. Helminth eggs (i.e., Capillarid, Strongyle, Oxyurid, Trematoda, Cestoda; Fig 7B and 7C) were detected in 2 *H*. *viridiflavus* and one *E*. *quatuorlineata*. One *H*. *viridiflavus* had a Trombiculidae mite larva (Table 4; Fig 7D).

**Table 4:** Parasitic forms observed through direct and/or flotation fecal tests with species of snake hosts.

Snake ID	Species	Sex	Direct fecal test	Flotation fecal test
CS007	*Zamenis longissimus*	M	Flagellates	-
CS011	*Hierophis viridiflavus*	M	Coccidia Capillarid eggs *Kalicephalus*-type eggs Trematode egg Acanthocephalan egg	Coccidia
CS021	*Zamenis longissimus*	M	Flagellates	-
CS025	*Elaphe quatuorlineata*	F	Flagellates	-
CS043	*Hierophis viridiflavus*	M	Flagellates	-
CS047	*Hierophis viridiflavus*	M	Flagellates	-
CS051	*Elaphe quatuorlineata*	F	Flagellates	-
CS078	*Hierophis viridiflavus*	M	-	*Nyctotherus* sp.
CS097	*Hierophis viridiflavus*	F	Flagellates	-
CS098	*Hierophis viridiflavus*	M	Capillarid eggs Trombiculidae mite larva	Capillarid eggs Cestode eggs
CS100	*Hierophis viridiflavus*	F	-	Sporulated *Eimeria*
CS102	*Elaphe quatuorlineata*	M	-	Oxyurid eggs
CS104	*Hierophis viridiflavus*	M	*Nyctotherus* sp.	-

**Fig 8:**
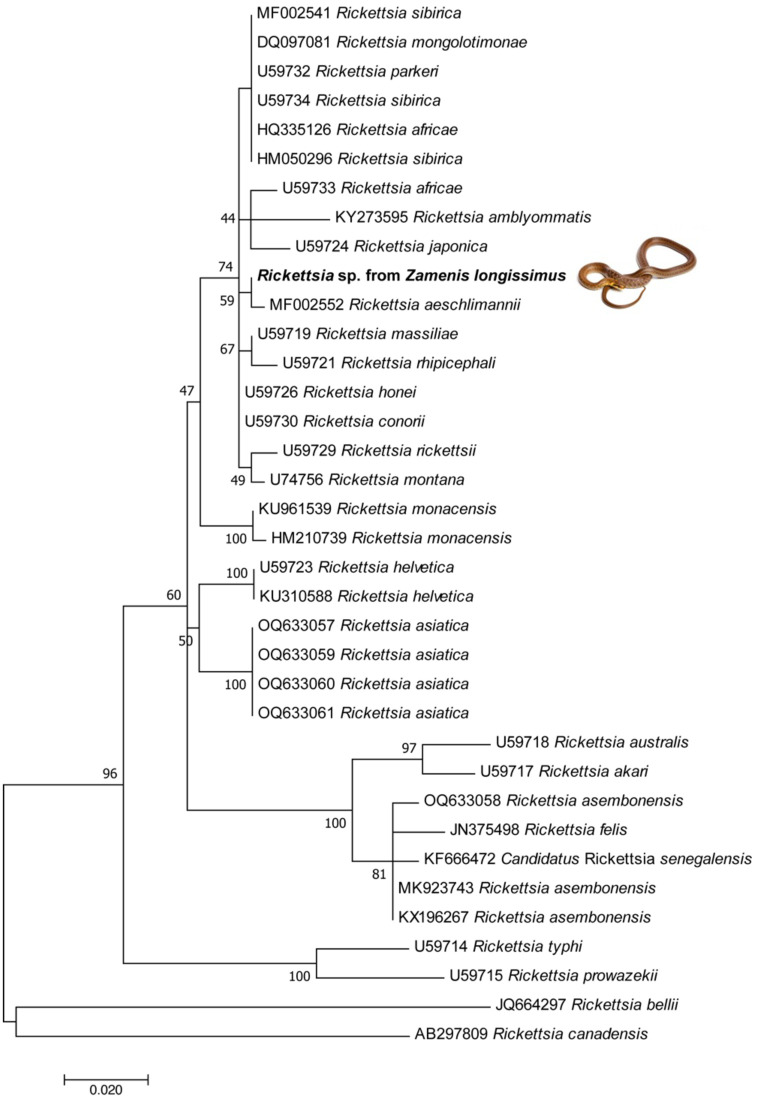
Maximum-likelihood phylogenetic trees of *gltA* genes of *Rickettsia* spp. Bootstrap values (>40%) are shown near the nodes. *Rickettsia belli*, *Rickettsia canadensis* were used as outgroups. Scale bar indicates nucleotide substitution per site. Sequences of this study are in bold.

**Fig 9:**
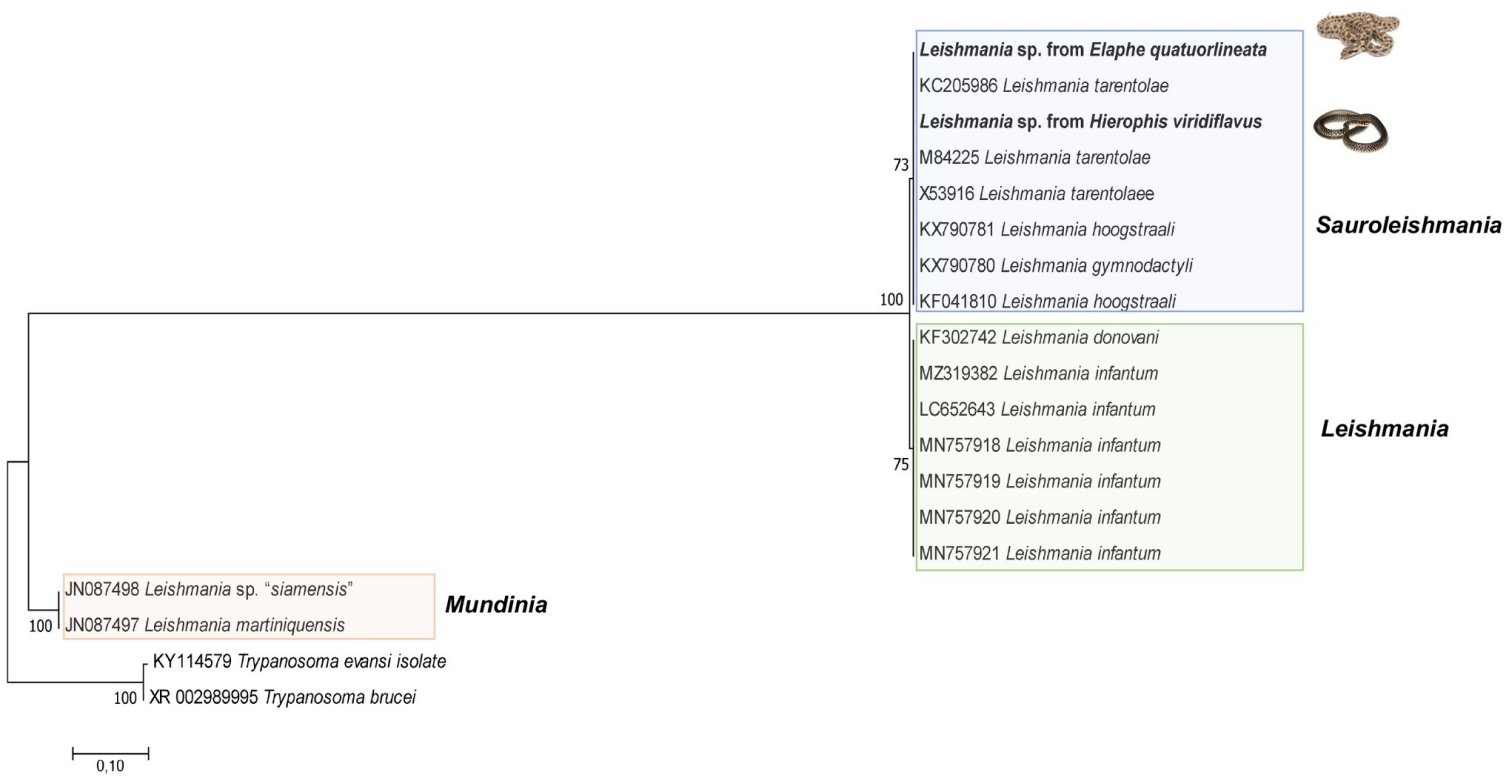
Maximum-likelihood phylogenetic trees of 18S rRNA genes of *Leishmania* spp. Bootstrap values (>40%) are shown near the nodes. *Trypanosoma brucei* and *Trypanosoma evansi* were used as outgroups. Scale bar indicates nucleotide substitution per site. Sequences of this study are in bold.

Overall, 28.5% (32/112) of snakes were molecularly positive for at least one microorganism or parasite (Table 5). Three *Ophionyssus* sp. of three different snake hosts (i.e., *E*. *quatuorlineata*, *H*. *viridiflavus*, *Z*. *longissimus*) were positive for the endosymbiont *Wolbachia* bacteria, similar to that detected in *Dermanyssus gallinae* from Japan (LC710644–97.62%) and from *Spinturnix* mites collected in bats from Thailand (KP165044–98.88%). In addition, one *Z*. *longissimus* snake was positive for *Rickettsia* (*gltA* gene) in blood, with high nucleotide identity (100%) to *Rickettsia aeschlimannii* detected in human blood from Kenya (RQB050057). Phylogenetic inference clustered the sequence of *Rickettsia* sp. generated in this study with *Rickettsia aeschlimannii* available in Genbank (Fig 8). On the other hand, *Leishmania tarentolae* was detected in cloacal swabs from two snakes (i.e., *E*. *quatuorlineata—*Ct 31,8; *H*. *viridiflavus—*Ct 31,65), whereas two *Leishmania* sp. 18S rRNA sequences were retrieved from the blood of snakes (i.e., *E*. *quatuorlineata*, *H*. *viridiflavus*) different from those positive in cloacal swabs. Phylogenetic inference, despite being discretely informative, clustered both sequences from this study with those of *Sauroleishmania* species, as well as clustering all the *Leishmania* subclade ones apart (Fig 9).

**Table 5:** Molecular identification of vector-borne and fecal pathogens detected in snakes.

Species of snake (Infected/total)	Vector-Borne Pathogen				Fecal pathogen		
	cPCR (16S rRNA)	cPCR (*glt*A)	cPCR (18S rRNA)	dqPCR (ITS1)	cPCR (*cox*1)	cPCR—nPCR (18S rRNA)	5plex-qPCR
*Coronella girondica* (0/1)							
*Elaphe quatuorlineata* (11/66)	(1)** *Wolbachia* sp.—LC710644 (97.62%)		(1)* Leishmania tarentolae - KC205986 (100%)	(1)*** Leishmania tarentolae (Ct 31,8)	(1)*** Oswaldocruzia filiformis - OQ346357 (87.96%)	(1)*** Alveolate FJ410512 (95.82%) (1)*** *Citrobacter freundii* CP026235 (99.23%) (1)*** Pseudomonas sp. CP043060 (93.75%)	(1)*** *Blastocystis* (2)*** Cryptosporidium (1)*** Giardia assemblage B
*Hierophis viridiflavus* (12/28)	(1)** *Wolbachia* sp.—LC710644 (97.40%)		(1)* Leishmania tarentolae - KC205986 (100%)	(1)*** Leishmania tarentolae (Ct 31,65)		(1)*** Achromobacter xylosoxidans HE798385 (97.77%) (1)*** Coccidia sp. MH590231 (88.7%) (1)*** Heteromita globosa - LC764482 (99.58%) (1)**** Pseudomonas brenneri LT629800 (98.65%)	(2)*** *Blastocystis* (2)*** Cryptosporidium (1)**** *Entamoeba*
*Natrix helvetica* (1/2)					(1)**** Proteus vulgaris—CP054157 (99.18%)		
*Zamenis longissimus* (8/15)	(1)** Wolbachia sp.—KP165044 (98.88%)	(1)* *Rickettsia aeschlimannii*—RQB050057 (100%)			(1)*** Rhabdias kafunata—OP605735 (89.46%)	(1)**** Pseudomonas brenneri LT629800 (97.32%) (1)*** Stenotrophomonas sp. CP109812 (99.77%) (1)*** Citrobacter braakii CP113163 (98.08%)	(2)*** Giardia assemblage B
**Total** (32/112)	(3/112)	(1/112)	(2/112)	(2/112)	(3/112)	(10/112)	(11/112)

*Blood;

**Mite;

***Swab;

****Feces

Furthermore, various fecal pathogens of zoonotic potential were detected by the 5plex qPCR, with high CT values (Table 5). In brief, *Blastocystis* spp. and *Cryptosporidium* spp. were detected in two species of snakes (i.e., *E*. *quatuorlineata*, *H*. *viridiflavus*), whilst *Entamoeba* spp. were detected in one *H*. *viridiflavus*, and *Giardia* assemblage B in two other species of snakes (i.e., *E*. *quatuorlineata*, *Z*. *longissimus*). The nPCRs for *Cryptosporidium* spp. and *Giardia* spp. confirmed the two *Cryptosporidum* spp., but did not give specific bands for *Giardia* spp. However, several sequences of bacteria were obtained from feces and cloacal swabs by sequencing un-specific bands of the two nPCRs (i.e., *Achromobacter xylosoxidans*, *Citrobacter braakii*, *Citrobacter freundii*, *Pseudomonas* sp., *Pseudomonas brenneri*, *Stenotrophomonas*) and protozoa (i.e., Alveolate, Coccidia, *Heteromita globosa*), one of the positive snakes was also positive to protozoa in the coprological tests (i.e., CS011—*H*. *viridiflavus*; Table 3). Additionally, *cox*1 sequences from the cloacal swab were obtained for two nematodes, one from an *E*. *quatuorlineata* similar to *Oswaldocruzia filiformis* from Russia (OQ346357–87.96%), and one from *Z*. *longissimus* similar to *Rhabdias kafunata* from China (OP605735–89.46). Moreover, a sequence was retrieved from a *N*. *natrix* with high homology to *Proteus vulgaris* from wastewater facilities in Canada (CP054157–99.18%; Table 5). Representative sequences herein generated were deposited in GenBank (accession number OR753376 for *gltA*; OR755903 to OQ630505 for 16S rRNA; OQ632771 to OQ632773, OR771463 to OR771475 and OR771476-OR771477 for 18S rRNA; OQ672452, OR758867, OR761977, OR761978 and for OR763078 *cox*1).

## Discussion

Ecto- and endoparasites were identified using a morpho-molecular approach from four of the five screened species of snakes collected for the “*festa dei serpari*” ritual. While most of the identified parasites are specific of reptiles and non-pathogenic (i.e., mites, helminths and protozoa), others transmitted by ticks (e.g., *Rickettsia*), as well as through fecal-oral transmission (i.e., *Cryptosporidium* spp., *Giardia*; *Pseudomonas*, *Proteus vulgaris*) have a zoonotic potential. The surveillance performed in this study with the local authorities, allowed to evaluate the parasitic fauna of free-ranging native snakes, which has been until now scarcely investigated or tackled in the Italian ophidic fauna.

The species composition of snake population (i.e., *E*. *quatuorlineata*, *H*. *viridiflavus*, *Z*. *longissimus*) is typical of the surroundings of Cocullo municipality, as already observed in previous screenings [27]. On the other hand, the absence of ticks in the snake population from this study may be due to the collection of the snakes during the early spring, where ophidians are less exposed to larvae and nymphs of *I*. *ricinus* [67]. Indeed, this tick species is most prevalent in woody areas of central Italy and was previously recorded in four-lined snakes from southern Italy [33]. In addition, immature stages of *I*. *ricinus* may prefer other reptile species that are more abundant and fossorial, such as *Podarcis* or *Lacerta* lizards [68,69]. The Ixodorhynchidae mites identified herein (*H*. *piger*) were described from an unknown snake collected in Florence (central Italy) [44]. Probably, *H*. *viridiflavus* is new host for this mite species, as well as Cocullo a new locality. The *Ophionyssus* species herein identified differs from the common snake mite, *O*. *natricis*, which is usually found in captive snakes around the world and may feed also on humans [70]. Moreover, the identified mite specimens morphologically differed from the 16 known species of *Ophionyssus* [42,43], with low homology in the *cox*1 nucleotide sequences compared to those of *O*. *natricis* deposited in Genbank. Given all the above, further studies are required to elucidate if the *Ophionyssus* species found is in fact a new species. Nonetheless, *H*. *viridiflavus* and *E*. *quatuorlineata* represent new hosts for *Ophionyssus* mites, as well as the Abruzzo region a new locality for Macronyssidae mites of snakes. Although all of the tested mites were negative for pathogens, the molecular detection of *Wolbachia* sp. in *Ophionyssus* sp. supports a previous finding in *O*. *natricis* from captive *Boa constrictor* [24], as well as in *Ornithonyssus bursa* [71]. The finding of *Wolbachia* in female mites, but not males, may be due to the role this endosymbiont bacterium displays in the reproduction of mites, through male-killing, feminization, and parthenogenesis [72,73].

Furthermore, the diversity and prevalence of the endoparasitic fauna of snakes recorded in this study (39.4%) were higher than that reported for captive snakes from Poland (i.e., 13.7%; [74]) and Italy (i.e., 10.5%; [23]), and lower from another survey conducted also in Italy (56.8%; [75]). Nevertheless, this study represents the first coprological survey of free-raging snakes in the Italian peninsula, without the need of euthanizing or working with recently dead/killed snakes [25,76,77]. Coccidia identified were morphologically similar with *Eimeria* [78], of mild pathogenicity, commonly observed in wild snakes. In addition, snakes were found infected with helminth eggs similar to *Kalicephalus*, *Rhabdias* and *Strongyloides* [79], as well as oxyurids and *Capillaria* eggs [80], with the latter being generally found in healthy animals. Overall, the findings of the above parasites may derive from the predation attitude of snakes [81] under different ecological contexts, therefore representing spurious parasites [82], rather than host specific ones. The possibility of having spurious parasites is higher in wild snakes than in those kept in captivity (e.g., 1.4%, 4/283; [82]), given that they actively feed on small preys. Given all the above, future studies are warranted to better distinguish real parasitic fauna of free-ranging snakes from spurious or free-living parasites. Importantly, molecular screening allowed to identify potentially zoonotic parasites and microorganisms, highlighting the need of an integrative approach using morpho-molecular techniques to assess wild animal populations under a One-Health perspective. For the zoonotic protozoa identified with the 5-plex qPCR, confirmatory sequences could only be retrieved for *Cryptosporidium*, but they were too short for reliable species identification. Zoonotic species, such as *Cryptosporidium muris*, *Cryptosporidium parvum* and *Cryptosporidium tyzzeri* were already identified in captive snakes [83]. Indeed, zoonotic *Cryptosporidium* species are spurious parasites in snakes, where resistant oocysts may contaminate the environment. Given that the diet of both species of snakes (i.e., *E*. *quatuorlineata*, *H*. *viridiflavus*) found positive for *Cryptosporidium* may include also rodents [84,85], further attempts should establish whether the captured snakes of Cocullo harbor zoonotic *Cryptosporidium* species or *Cryptosporidium serpentis*. The latter species may cause asymptomatic or chronic infections, being highly pathogenic, infectious, and irresponsive to therapeutic treatment [86]. The 5-plex qPCR may have low specificity and limited discriminatory power for the highly diverse protozoa species in snakes’ feces [62,87]. Moreover, mixed infections with related species of protozoa result in close melting curves hindering the detection of targeted species. Nonetheless, specific investigations on zoonotic pathogens in snakes are warranted considering that *G*. *duodenalis* is an important food and waterborne pathogen [88] as well as *E*. *histolytica*. The latter has never been reported in snakes [89], which typically host a highly pathogenic reptilian protozoan, *Entamoeba invadens*, causing necrotic enteritis and hepatitis [90], as well as the less pathogenic species *Entamoeba ranarum* [90]. Certainly, the integrative approach using morphological and molecular tools further permitted the identification of nematodes belonging to the genera *Oswaldocruzia* and *Rhabdias* from cloacal swabs, when fecal samples were not collected [91,92]. These genera of nematodes are potentially pathogenic to snakes and should be actively surveyed to assess their deleterious effects on the free-raging snake populations, also considering the zoonotic potential and emergence of snake-associated human parasites, such as *Ophidascaris robertsi* neural *larva migrans* [93].

While it was not possible to perform a definitive identification of zoonotic parasites, the sequences obtained from fecal and cloacal swabs allowed the detection of zoonotic bacteria (i.e., *A*. *xylosoxidans*, *C*. *freundii*, *P*. *vulgaris*, *Pseudomonas*), which were already detected in snakes, that may act both as reservoirs and spreaders [94,95]. As the bacteria above may be opportunistic pathogens of humans and multi-drug resistant strains have been identified [96], correct biosafety measures should be applied during and after the “*festa dei serpari*” event. This is mainly due to the fact that, when handled, free-ranging snakes defecate as a defense mechanism [97,98], therefore increasing the risks of contamination with pathogens such as *Salmonella* [29,99].

Regarding zoonotic vector-borne pathogens, the detection of *R*. *aeschlimannii* in *Z*. *longissimus* blood suggests exposure to tick bites, further corroborating the potential role of reptiles as reservoirs for *Rickettisa* spp. [100,101]. Again, molecular positivity for species of the spotted fever group (i.e., *Rickettsia monacensis*, *Rickettsia helvetica*) was reported in lizards from Italy [35] and snakes (i.e., *Rickettsia asiatica*) from Morocco [3]. As *R*. *aeschlimannii* and other rickettsiae (i.e., *R*. *monacensis*, *Rickettsia massiliae*) are considered as emerging human pathogens [102], further studies should be conducted to verify the occurrence of this species of *Rickettsia*, previously detected in Algeria from *Hyalomma aegyptium* ticks collected from tortoises [103].

The retrieval of the reptile-associated *L*. *tarentolae* from snakes in Italy represents new hosts (i.e., *E*. *quatuorlineata*, *H*. *viridiflavus*) and broadens its geographical distribution northwards, near the Lazio region where it was previously detected in human blood donors and sand flies [104]. Prior surveys from Italy yielded positive molecular results in species of lizards and geckos for *L*. *infantum* and *L*. *tarentolae*, in urban, peri-urban areas and dog shelters [105]. In addition, *L*. *tarentolae* was detected for the first time from cloacal swabs, however, attempts to isolate *Leishmania* spp. from snakes are warranted given that, thus far *L*. *tarentolae* has been isolated only from geckos of the Mediterranean basin [106,107]. To further address the epidemiological picture of *Leishmania*, entomological surveys are pivotal to describe the species composition of sand flies and address if there is also a sympatric occurrence of both *L*. *tarentolae* and *L*. *infantum* in the surrounding of the Cocullo municipality, given that *L*. *tarentolae* can potentially infect mammals (i.e., humans and dogs) [106].

Given all of the above, the population of snakes around Cocullo may be in part under a “positive” human-snake relationship, where traditional beliefs impact directly on reptile conservation. This, effect has been already studied in other cultural contexts for reptiles such as water monitor lizards in the surroundings of a small village in northern India [108]. As in Cocullo, villagers from the small town of Chak Manik have many beliefs (i.e., protecting the marshlands for their Gods and for the village to thrive), that indirectly have a positive effect on the vulnerable population of reptiles, being mutually beneficial for both the villagers and the reptile species [108]. Although highly subjective, overall health status and condition of the screened snakes was established as “apparently healthy”. Compared to the previous study, where 23 animals had some type of dermal abnormality [27], the three snakes that were herein reported as having skin lesions, were all probably signs of healed trauma or infection. However, given that it was not possible to discard fungal granuloma, annual screening of the collected snake population of Cocullo, assessing fungal, bacterial, and parasitic infections, should be encouraged to have a well-established and consistent surveillance program that will allow for rapid detection of harmful and zoonotic pathogens. Accordingly, results from this study will aid to create strategies to prevent zoonotic transmission of pathogens. Indeed, alongside the established snakes’ population monitoring efforts, pathogens surveillance using a multi-sectorial approach should be also performed annually to assess zoonotic pathogen emergence and their dynamics within the capture population of snakes, that are after released in the environment [109]. Data generated from the present study will be useful for local and national authorities to formulate proper prevention policies specific for the *serpari* as well as for tourist and pilgrims that participate in the event. For example, education and training of *serpari* on proper husbandry and protective measures when capturing and handling snakes will aid to reduce the risk of transmission of pathogens to this group of people that are at higher risk, as they are in contact with snakes for over a month [110]. Indeed, educating *serpari* on proper husbandry and procedures such as quarantine of snakes and the proper use of personal protective equipment (PPE), may reduce the risk of transmission from snake to snake, as well to humans [111]. On the other hand, coordinated policies with local authorities during the event are important to minimize the risk of oral-fecal transmission of pathogens from snakes to tourists. These prevention strategies should be focused on providing hand hygienization/disinfection places, as well as information on why and how to wash their hand after handling a wild animal [112]. Using a One-Health approach to monitor the snake population and reduce the risk of zoonotic transmission will thereby contribute to the conservation of the snakes and the perpetuation of the tradition.

## Conclusion

Data presented here demonstrate that using an ethnoherpetoparasitological framework to assess the collected ophidian population prior to the annual celebration of the “*festa dei serpari*” ritual in Cocullo, is a useful approach that allowed for the assessment of the health of the handled snakes, as well as the risks of transmission of zoonotic pathogens present in wild populations. Although snake collected for the ritual harbored reptile-specific and non-pathogenic mites, helminths, and protozoa, the presence of zoonotic pathogens should not be disregarded. This is the case with vector-borne pathogens (e.g., *Rickettsia*, *Leishmania*), as well as opportunistic zoonotic pathogens (i.e., *Cryptosporidium*, *Giardia*, *A*. *xylosoxidans*, *C*. *freundii*, *P*. *vulgaris*, *Pseudomonas*) present in the feces of these scaley animals. Thus, snakes collected and showcased in the “*festa dei serpari”* are optimal sentinels and bioindicators of environmental and ophidian population health, as well as reservoirs of microorganisms that should be controlled through proper biosafety measures when handled by *serpari* to avoid the risk of zoonotic transmission. Effective public health policies within this unique epidemiological context are advocated, while promoting targeted conservation initiatives, education, and biosafety measures.

## Supporting information

S1 Video *Festa dei Serpati* ritual. Every 1^st^ of may for more than 500 hundred years, the statue of *San Domenico* is covered with four-lined snakes (*Elaphe quatuorlineata*), followed by a procession through the small town of Cocullo, Italy.
(MP4)
